# Concurrent Functional Linear Regression Via Plug-in Empirical Likelihood

**DOI:** 10.1007/s13171-024-00369-3

**Published:** 2024-09-02

**Authors:** Hsin-wen Chang, Ian W. McKeague

**Affiliations:** 1 Academia Sinica, Taipei, Taiwan; 2 Columbia University, New York, NY USA; 3 City University of Hong Kong, Hong Kong, China

**Keywords:** Accelerometry, Bootstrap, Functional data analysis, Nonparametric likelihood ratio, Primary: 62R10, Secondary: 62G15, 62J99

## Abstract

Functional data with non-smooth features (e.g., discontinuities in the functional mean and/or covariance) and monotonicity arise frequently in practice. This paper develops simultaneous inference for concurrent functional linear regression in this setting. We construct a simultaneous confidence band for a functional covariate effect of interest. Along with a Wald-type formulation, our approach is based on a powerful nonparametric likelihood ratio method. Our procedures are flexible enough to allow discontinuities in the coefficient functions and the covariance structure, while accounting for discretization of the observed trajectories under a fixed dense design. A simulation study shows that the proposed likelihood ratio-based procedure outperforms the Wald-type procedure in moderate sample sizes. We apply the proposed methods to studying the effect of age on the occupation time curve derived from wearable device data obtained in an NHANES study.

## Introduction

It has been of great interest to see how certain predictors can be related to physical activity in observational studies (Sallis, 2000). Nowadays, physical activity is often assessed by wearable device measurements (Wright et al., 2017). Chang and McKeague (2022a) develop inference for the mean of occupation time curves constructed from such measurements; these curves give the amount of time that a sensor reading exceeds a given activity level as that level varies. In the presence of jump discontinuities and monotonicity of such curves and their moments, the use of existing functional data methods can result in a loss of information if the presence of those features is ignored. To our knowledge, there have not been any simultaneous inference methods for functional regression that can incorporate such features.

To bridge this gap in the literature, we develop simultaneous confidence bands for concurrent functional linear (CFL) regression (Ramsay and Silverman, 2005) based on the framework in Chang and McKeague (2022a). In particular, we can handle discontinuities of the functional data and their moments, as well as non-smooth effects between the covariate and response functions. Further, the empirical likelihood (EL) approach is used to incorporate monotonicity information and to achieve optimality (for example, in terms of Bahadur efficiency; see Remark 5 after Theorem 2 for more details). Notably, it turns out that the conditions we need are weaker than existing conditions imposed when using smoothing to carry out simultaneous inference for CFL models under a fixed dense design (see the next paragraph for a literature review) (Degras, 2011; Cao et al., 2012). This allows us to deal with a wider range of functional data (beyond occupation time curves), such as curves with non-smooth latent mean and covariance functions, or non-smooth regression relationship (see Section 3 for an illustration). We focus on building a confidence band for one covariate effect of interest while adjusting for the other covariates.

While there are many methods available for the estimation (see, e.g., Ghosal et al. 2020, and the references therein) and simultaneous testing (Wang et al., 2018; Ghosal and Maity, 2022) for the regression coefficient functions in CFL models under a fixed dense design, there are relatively few procedures for constructing simultaneous confidence bands for these functions. Note that going from simultaneous testing to simultaneous confidence bands may not be as simple as inverting a statistical test in traditional statistics, because the simultaneous test statistic will need to be a maximally selected function of the true regression coefficient functions, and to our knowledge such statistics have not been used in the aforementioned simultaneous testing. Cao et al. (2020) considered a more general function-on-function regression and derived simultaneous confidence bands based on polynomial spline smoothing. However, they assumed continuity of at least the first-order partial derivatives for the coefficient functions, whereas we allow discontinuities in these functions.

The use of EL in CFL models has been considered in Wang et al. (2018), but the confidence interval they constructed for the covariate effect of interest is not simultaneous. Furthermore, their procedure requires smoothing and a profile likelihood approach. In contrast, we do not require smoothing, and we will use plug-in estimates for those regression coefficient functions we deem as less important; EL with plug-in has been recommended for handling infinite-dimensional nuisance parameters due to its computational tractability (Hjort et al., 2009).

The paper is organized as follows. The Wald-type and the EL-based confidence bands are developed in Sections 2.1 and 2.2, respectively. Section 3 presents simulation results showing that proposed EL-based procedure outperforms the Wald-type procedure. In Section 4 we analyze an example based on NHANES data, and Section 5 provides discussion. Proofs are presented in the Appendices.

## Method

### Functional Linear Regression for Discretized Observations

In this section, we first describe the underlying trajectories that generate the functional data, and the relationship between such latent predictors and response. Then we define a discretization mechanism that represents the lens through which we observe the underlying trajectories, and investigate the properties of an interpolated version of the least-squares estimator (LSE) of the regression coefficient function of interest.

Let $\{Y_i(a), \boldsymbol{X}_i(a)\}$, i=1,…,n and $a \in [\alpha _1, \alpha _2]\}$, be *n* i.i.d. realizations of a measurable stochastic process $\{Y(a), \boldsymbol{X}(a)\}$ having right-continuous sample paths of bounded variation. The relationship between *Y*(*a*) and $\boldsymbol{X}(a)$ is given by

$$ E\{Y(a) | \boldsymbol{X}(a)\} = \boldsymbol{\beta }^T(a)\boldsymbol{X}(a), $$

where $\boldsymbol{\beta }(a) = (\beta _1(a), \ldots , \beta _p(a))^T$ is a *p*-dimensional vector of unknown coefficient functions. Suppose that $E\left( \boldsymbol{X} \boldsymbol{X}^T\right) (a)$ has full rank *p* for all $a \in [\alpha _1, \alpha _2]$. Note the error process $\varepsilon (a) = Y(a) - \boldsymbol{\beta }^T(a)\boldsymbol{X}(a)$ has mean 0 due to the above display, but we do not need to assume that it is independent of $\boldsymbol{X}(a)$ as in the existing literature (see, e.g., Wang et al. 2018, for such an assumption in EL-based tests in a concurrent linear model for functional data). Let $\hat{\boldsymbol{\beta }}(a)$ be the LSE of $\boldsymbol{\beta }(a)$ at each $a \in [\alpha _1, \alpha _2]$; that is, $\hat{\boldsymbol{\beta }}(a) = \{\sum _{i = 1}^n (\boldsymbol{X}_i \boldsymbol{X}^T_i)(a) \}^{-1}\{\sum _{i = 1}^n (\boldsymbol{X}_i Y_i)(a)\}$. Instead of fully observed trajectories (of the stochastic process $\{Y(a), \boldsymbol{X}(a)\}$), we can only observe $\{Y(a), \boldsymbol{X}(a)\}$ on $\textbf{G}_n$, a (not necessarily equispaced) grid of points in $[\alpha _1,\alpha _2]$ (including the endpoints). Denote the discretized observation as $\{\textrm{f}_n(Y)(a), \textrm{f}_n(\boldsymbol{X})(a)\}$, and $\textrm{f}_n(\boldsymbol{\beta })$ and $\textrm{f}_n(\hat{\boldsymbol{\beta }})$ the corresponding coefficient function and sample coefficient function, respectively. Here $\textrm{f}_n(\boldsymbol{\phi })$ is the discretization of some real-, vector- or matrix-valued function $\boldsymbol{\phi }$ over $[\alpha _1,\alpha _2]$ defined by

1 $$\begin{aligned} \textrm{f}_{n}(\boldsymbol{\phi })(a) = {\left\{ \begin{array}{ll} \boldsymbol{\phi }(a), & a \in { \textbf{G}}_n \\ \boldsymbol{\phi }(b_a), & a \in [\alpha _1,\alpha _2] \setminus { \textbf{G}}_n, \end{array}\right. } \end{aligned}$$

and $b_a$ is the closest point on the grid to the right of *a*. This discretization $\textrm{f}_n(\boldsymbol{\phi })$ transforms the complete trajectory of $\boldsymbol{\phi }$ into a vector or matrix of step functions. As is typical in dense functional data analysis settings (Wang et al., 2018), the mesh of $\textbf{G}_n$ (the maximal distance between adjacent grid points) is assumed to shrink more quickly than a certain negative power of *n* as $n \rightarrow \infty$. Further, we need a condition involving the right-hand γ-Dini derivatives (Chang and McKeague, 2022a):

$$\begin{aligned}&D^+(\beta _j, \gamma )(a) = \limsup _{h \rightarrow 0+} \frac{\beta _j(a+h)-\beta _j(a)}{h^\gamma }\text{, } \text{ and } \\ &D_+(\beta _j, \gamma )(a) = \liminf _{h \rightarrow 0+} \frac{\beta _j(a+h)-\beta _j(a)}{h^\gamma } \end{aligned}$$

for γ>0 and j=1,…,p. These γ-Dini derivatives always exist in $[-\infty , \infty ]$. They are reminiscent of γ-Hölder continuity, but our requirement of boundedness of the right-hand γ-Dini derivatives in the following theorem is much weaker than γ-Hölder continuity.

The following theorem describes the asymptotic behavior of the estimated coefficient function based on the aforementioned discretization. Here and in the sequel, the convergence in distribution (denoted by ${\buildrel {d} \over \longrightarrow }$) of a sequence of random elements in a metric space $\mathbb {D}$ means convergence of the expectation of every bounded, continuous real-valued function applied to each element of the sequence (see, e.g., van der Vaart 2000, page 258). In the following theorem we use $\mathbb {D} =\ell ^\infty ( [\alpha _1,\alpha _2])$, the space of all bounded real-valued functions on $[\alpha _1,\alpha _2]$, endowed with the supremum norm.

For the theorem below, define $X_j(a)$ to be the *j*-th element of $\boldsymbol{X}(a)$, and $K(a, b) = K(a, b | \boldsymbol{X}) =\textrm{Cov}\{\varepsilon (a),\varepsilon (b) | \boldsymbol{X}\}$ represents a possibly heteroscedastic covariance function that depends on $\boldsymbol{X}$. The proof of the theorem is in Appendix A.

#### Theorem 1

Suppose the sample paths of $X_j(a)$ and ε(a) are right-continuous, have bounded variation, and are uniformly bounded by some finite constant τ. Also assume that $E(X_j\varepsilon )(\cdot )$ and $E(X_jX_{\ell })(\cdot )$ have at most finitely many jump discontinuities, $j, \ell = 1, \ldots , p$, and $\inf _{a \in [\alpha _1,\alpha _2]} \det \{E(\boldsymbol{X} \boldsymbol{X}^T)\}(a) > 0$. Then, if the mesh of $\textbf{G}_n$ is *o*(1), for all sufficiently small δ>0 there exists $I_\delta \subset [\alpha _1,\alpha _2]$ having Lebesgue measure δ such that

$$\sqrt{n} \left\{ \textrm{f}_{n}(\hat{\boldsymbol{\beta }}) - \textrm{f}_{n}(\boldsymbol{\beta }) \right\} (a) {\buildrel {d} \over \longrightarrow }{\boldsymbol{U}}(a)$$

in $\ell ^\infty ( [\alpha _1,\alpha _2]\setminus I_\delta )$ as $n \rightarrow \infty$, where ${\boldsymbol{U}}(a)$ is a *p*-variate Gaussian process with zero mean and covariance function $\textrm{Cov}\{{\boldsymbol{U}}(a),{\boldsymbol{U}}(b)\}$ given by

$$\begin{aligned} \left\{ E\left( \boldsymbol{X} \boldsymbol{X}^T\right) \right\} ^{-1}(a) E\left\{ K(a, b) \boldsymbol{X}\left( a\right) \boldsymbol{X}^T\left( b\right) \right\} \left\{ E\left( \boldsymbol{X} \boldsymbol{X}^T\right) \right\} ^{-1}(b),\ a, b \in [\alpha _1, \alpha _2]. \end{aligned}$$

Suppose, in addition, that $D^+(\beta _j, \gamma )(a)$ and $D_+(\beta _j, \gamma )(a)$ are bounded over $a \in [\alpha _1,\alpha _2]$ for some γ>0, and that $\beta _j(a)$ is right-continuous, of bounded variation, and has at most finitely many jump discontinuities, j=1,…,p. Then, if the mesh of $\textbf{G}_n$ is $o(n^{-1/(2\gamma )})$,

$$\sqrt{n} \left\{ \textrm{f}_{n}(\hat{\boldsymbol{\beta }}) - \boldsymbol{\beta }\right\} (a) {\buildrel {d} \over \longrightarrow }{\boldsymbol{U}}(a)$$

in $$\ell ^\infty ( [\alpha _1,\alpha _2]\setminus I_\delta )$$ as $$n \rightarrow \infty $$.

#### Remark 1

Note that if we assume homoscedasticity (i.e. *K*(*a*, *b*) does not depend on $\boldsymbol{X}$), then the covariance function $\textrm{Cov}\{{\boldsymbol{U}}(a),{\boldsymbol{U}}(b)\}$ becomes

$$\begin{aligned} K(a, b) \left\{ E\left( \boldsymbol{X}_i \boldsymbol{X}_i^T\right) \right\} ^{-1}(a)E\left\{ \boldsymbol{X}_i\left( a\right) \boldsymbol{X}_i^T\left( b\right) \right\} \left\{ E\left( \boldsymbol{X}_i \boldsymbol{X}_i^T\right) \right\} ^{-1}(b) \end{aligned}$$

for $a, b \in [\alpha _1, \alpha _2]$, and reduces to

$$ K(a, a) \left\{ E\left( \boldsymbol{X}_i \boldsymbol{X}_i^T\right) \right\} ^{-1}(a), $$

the inverse of the Fisher information matrix at *a*, when a=b.

#### Remark 2

By a similar reasoning as in Section 11 of Chang and McKeague (2022b), note that the right-continuity condition can be replaced by left-continuity, with $b_a$ in the definition of $f_n(g)(a)$ changing to the closest point on $\textbf{G}_n$ to the left of *a*, and limits changing to left-hand ones instead in defining the γ-Dini derivatives.

#### Remark 3

The first part of Theorem 1 holds irrespective of how quickly the mesh of $\textbf{G}_n$ shrinks as $n \rightarrow \infty$. Thus, there is no distinction between moderately dense and dense functional data here (Wang et al., 2018). Such a distinction only matters in the second part of the theorem, which can be used to construct a simultaneous confidence band for $\beta (\cdot )$ for *essentially all* $a\in [\alpha _1,\alpha _2]$ (Chang and McKeague 2022b, Section 2.1), provided the mesh of $\textbf{G}_n$ tends to zero faster than $n^{-1/(2\gamma )}$.

#### Remark 4

The first part of the theorem applies to occupation time curves as the response or covariate functions in CFL regression, because the bounded variation, uniform bound and right-continuity conditions are satisfied due to the monotonicity, boundedness and right-continuity of occupation time curves (Chang and McKeague 2022a, Section 2.1), respectively. The second part of the theorem applies to occupation time curves as the response functions for γ=1, because the instantaneous change in the mean occupation time function from the right is bounded between 0 and the total study time (Chang and McKeague 2022a, Section 2.1).

The distribution of the limiting process ${\boldsymbol{U}}(a)$ needs to be estimated because it is not distribution-free. This can be done using the paired bootstrap (Chatterjee and Bose, 2005) $\textrm{f}_n(U_n^*)(a)$ based on sampling *n* curves with replacement from $\{\textrm{f}_n(\boldsymbol{X}_1\hat{\varepsilon }_1)(a), \ldots , \textrm{f}_n(\boldsymbol{X}_n\hat{\varepsilon }_n)(a), a \in [\alpha _1, \alpha _2]\}$, where $U_n^*(a) = \sqrt{n} \{ \hat{\boldsymbol{\beta }}^*(a) - \hat{\boldsymbol{\beta }}(a)\}$, $\hat{\boldsymbol{\beta }}^*(a) = \left\{ \sum _{i = 1}^n (\boldsymbol{X}_i \boldsymbol{X}_i^T)/ n\right\} ^{-1}(a) \sum _{i=1}^{n} \{W_{ni} \boldsymbol{X}_i(a) \hat{\varepsilon }_i(a)\} /n$, $\hat{\varepsilon }_i(a) = Y(a) - \hat{\boldsymbol{\beta }}^T(a)\boldsymbol{X}(a)$, and $W_{ni}$ is the number of times that $\textrm{f}_n(\boldsymbol{X}_i\hat{\varepsilon }_i)(a)$ is redrawn.

Bootstrap consistency of $\textrm{f}_n(U_n^*)(a)$ is established as follows (see Appendix B for the proof). Interestingly, in constrast to the different conditions needed in the two parts of Theorem 1, this bootstrap consistency result holds irrespective of how quickly the mesh shrinks.

#### Corollary 1

Under the conditions of the first part of Theorem 1, for all sufficiently small δ>0, there exists $I_\delta \subset [\alpha _1,\alpha _2]$ having Lebesgue measure δ such that $\textrm{f}_n(U_n^*)$ converges weakly to ${\boldsymbol{U}}(a)$ in $\ell ^\infty ( [\alpha _1,\alpha _2] \setminus I_\delta )$ as $n \rightarrow \infty$, given the data sequence $\{\textrm{f}_j(Y_i), \textrm{f}_j(\boldsymbol{X}_i), i=1,\ldots , j, j=1, 2, \ldots \}$, in probability.

From this result, according to Theorem 1, we can construct an asymptotic $100(1-\alpha )\%$ simultaneous confidence band for $\beta (\cdot )$ as $\textrm{f}_n(\hat{\boldsymbol{\beta }})(a) \pm n^{-1/2} c^*_{NS, \alpha }$ for essentially all $a\in [\alpha _1,\alpha _2]$, where $c^*_{NS, \alpha }$ denotes the upper α-quantile of the $\sup _{a \in [\alpha _1,\alpha _2]} \left| \textrm{f}_n(U_n^*(a))\right|$ values obtained from *B* bootstrap samples; we use B=1000 for implementation. We refer to this as the Wald-type *NS band*, where NS stands for “non-standardized”, which corresponds to the NS band in Chang and McKeague (2022a) when X(a)≡1 and p=1. Unfortunately, this band did not perform well in our simulation study (see Section 3), because it does not have the optimality EL enjoys. An alternative approach is developed in Section 2.2.

### Empirical Likelihood Confidence Band for Each Regression Coefficient

In this section, we develop the proposed confidence band for each of the regression coefficients $\beta _j(\cdot )$, j=1,…,p. Our approach is based on inverting a localized form of the EL statistic at each value of *a*. For simplicity of exposition, we define the observed EL ratio by discretizing the fully observed trajectories of the EL ratio process in the following, instead of defining the discretized verion of each component that determines the EL ratio. But note that those components are available to us only in their discretized forms.

For a given $a \in [\alpha _1,\alpha _2]$, the local EL ratio for $\beta _j(a)$ is $\textrm{f}_n(\mathcal {R}(\tilde{\beta }_j))(a)$ at a given value $\tilde{\beta }_j(a)$, where $\mathcal {R}(\tilde{\beta }_j)(a)$ is

2 $$\begin{aligned} \frac{ \sup { \left\{ L(P_{a}): P_{a}\left\{ \boldsymbol{x}\left( y - \sum _{\ell \ne j}\hat{\beta }_{\ell }(a) x_{\ell } - \tilde{\beta }_j(a) x_j \right) \right\} = 0, P_{a} \in \Gamma _{a} \right\} } }{\sup { \left\{ L(P_{a}): P_{a} \in \Gamma _{a} \right\} } }, \end{aligned}$$

$P_{a}(\cdot )$ is a candidate for the distribution of $\{Y(a), \boldsymbol{X}(a)\}$, $P_{a}h$ is an abbreviation of $\int h dP_{a}$ for a given measurable function *h* of $(y, \boldsymbol{x}) \in \mathbb {R} \times \mathbb {R}^p$, $\hat{\beta }_j(a)$ is the *j*-th element of $\hat{\boldsymbol{\beta }}(a)$, $\Gamma _{a}$ is the set of distributions supported by $\{Y_i(a), \boldsymbol{X}_i(a)\}_{i=1}^n$, $L(P_{a})=\prod _{i=1}^n P_{a}(\{Y_i(a), \boldsymbol{X}_i(a)\})$ is the nonparametric likelihood, and we follow the convention $\sup \emptyset = 0$. Note that in formulating the local EL ratio for each regression coefficient, we treat the other regression coefficients as nuisance parameters and replace them with plug-in LSEs.

We now state our first key result, giving the asymptotic distribution of the EL statistic $-2\log \textrm{f}_n(\mathcal {R}(\boldsymbol{\beta }))(a)$ viewed as a process indexed by *a*. Let $\boldsymbol{\sigma }^2(a) = E\left\{ K(a, a) \boldsymbol{X}\left( a\right) \boldsymbol{X}^T\left( a\right) \right\}$ and $\boldsymbol{g}$ be the projection operator on $(\ell ^\infty ( [\alpha _1,\alpha _2]))^{p}$ given by

$$ \boldsymbol{g}\left( \boldsymbol{e}\right) \left( a\right) = \boldsymbol{e}\left( a\right) - E\left( {\boldsymbol{X}} {\boldsymbol{X}}^T_{(-j)}\right) \left( a\right) \left\{ E\left( \boldsymbol{X} \boldsymbol{X}^T\right) \right\} ^{-1}_{(-j)}\left( a\right) \boldsymbol{e}\left( a\right) , $$

where for a vector/matrix ${\boldsymbol{v}}$, we use ${\boldsymbol{v}}_{(-j)}$ to denote the vector/matrix after removing the *j*-th element/row of ${\boldsymbol{v}}$.

#### Theorem 2

Suppose the conditions of Theorem 1 hold and in addition, $\inf _{a \in [\alpha _1,\alpha _2]}\det {\{\boldsymbol{\sigma }^2(a)\}} > 0$ and $\boldsymbol{\sigma }^2(\cdot )$ has at most finitely many jump discontinuities. Then for all sufficiently small δ>0, there exists $I_\delta \subset [\alpha _1,\alpha _2]$ having Lebesgue measure δ such that

$$ -2\log f_n\left( \mathcal {R}(\beta _j)\right) \left( a\right) {\buildrel {d} \over \longrightarrow }\boldsymbol{g}\left( \boldsymbol{E}\right) ^T \left( a\right) \boldsymbol{\sigma }^{-2}\left( a\right) \boldsymbol{g}\left( \boldsymbol{E}\right) \left( a\right) $$

in $\ell ^\infty ( [\alpha _1,\alpha _2]\setminus I_{\delta })$ as $n \rightarrow \infty$, where ${\boldsymbol{E}}(a)$ is a tight *p*-variate Gaussian process with zero mean and covariance function $E\left\{ K(a, b) \boldsymbol{X}\left( a\right) \boldsymbol{X}^T\left( b\right) \right\}$.

#### Remark 5

The optimality properties of EL applicable to our (pointwise) regression settings have been obtained in terms of (1) the large deviation principle (Kitamura, 2007; Kitamura et al., 2012), and (2) Bahadur efficiency (Bahadur, 1967; Otsu, 2010). EL enjoys these optimality properties while other procedures (even the first-order asymptotically equivalent ones) may not.

#### Remark 6

The proof (given in Appendix C) is based on a uniform approximation of the EL statistic by $\textrm{f}_n(\hat{\Uppsi })(a)$, where $\hat{\Uppsi }(a)= \boldsymbol{g}(\boldsymbol{E}_n)^T \boldsymbol{\sigma }^{-2}(a) \boldsymbol{g}(\boldsymbol{E}_n)$ and ${\boldsymbol{E}}_n(a) = \sum _{i = 1}^n (\boldsymbol{X}_i\varepsilon _i)(a) / \sqrt{n}$. Note, however, that the asymptotic equivalence of $\textrm{f}_n(\hat{\Uppsi })(a)$ to the EL statistic does not imply optimality of $\textrm{f}_n(\hat{\Uppsi })(a)$, as mentioned in the previous remark.

#### Remark 7

The condition $\inf _{a \in [\alpha _1,\alpha _2]}\det {\{\boldsymbol{\sigma }^2(a)\}} > 0$ is similar to the condition of a positive definite information or covariance matrix in the Wilks type theorem (Owen, 2001). To deal with data that violate this condition, we adapt a two-step approach that has been proposed in Nair (1984) and Section 5.1 of Chang and McKeague (2022b). More specifically, we first construct the prescribed band up to a certain point $\hat{r} = \hat{r}(z)$ in terms of z∈[0,1], where $\hat{r}=\sup \{a: \inf _{a \in [\alpha _1,\alpha _2]}\det {\{\hat{\boldsymbol{\sigma }}^2(a)\}} > z \}$; z=0.05 has worked well in the literature. Second, use a principled approach as follows to extend the confidence band beyond $\hat{r}$: for the upper/lower boundaries of the confidence bands beyond the right endpoint, we use the upper/lower boundaries of the NS band.

For calibration, we use a similar nonparametric bootstrap method as in Section 2.1, based on sampling *n* curves with replacement from $\{\textrm{f}_n(\boldsymbol{X}_1\hat{\varepsilon }_1)(a), \ldots , \textrm{f}_n(\boldsymbol{X}_n\hat{\varepsilon }_n)(a), a \in [\alpha _1, \alpha _2]\}$. Since $M_n = \sup _{a\in [\alpha _1,\alpha _2]}$ $\{-2\log \textrm{f}_n(\mathcal {R}(\beta _j))(a)\}$ is asymptotically equivalent to $\sup _{a\in [\alpha _1,\alpha _2]} \textrm{f}_n(\hat{\Uppsi })(a)$ by the above Remark 6, it suffices to bootstrap $\textrm{f}_n(\hat{\Uppsi })(a)$ by $\textrm{f}_n(\hat{\Uppsi }^{*})(a)$, where $\hat{\Uppsi }^{*}(a) = \hat{\boldsymbol{g}}(\boldsymbol{E}_n^*)^T \hat{\boldsymbol{\sigma }}^{-2}(a) \hat{\boldsymbol{g}}(\boldsymbol{E}_n^*)$, ${\boldsymbol{E}}_n^*(a) = \sum _{i = 1}^n \{(W_{ni}-1)(\boldsymbol{X}_i\hat{\varepsilon }_i)(a)\} / \sqrt{n}$, $\hat{\boldsymbol{g}}$ be the projection operator on $(\ell ^\infty ( [\alpha _1,\alpha _2]))^{p}$ given by

$$ \hat{\boldsymbol{g}}\left( \boldsymbol{e}\right) \left( a\right) \!=\! \boldsymbol{e}\left( a\right) \!-\! \left\{ \!\sum _{i = 1}^n \left( \boldsymbol{X}_i \boldsymbol{X}_{i, (-j)}^T\right) / n\!\right\} \left( a\right) \left\{ \!\sum _{i = 1}^n \left( \boldsymbol{X}_i \boldsymbol{X}_i^T\right) / n\!\right\} ^{-1}_{(-j)}\left( a\right) \boldsymbol{e}\left( a\right) , $$

and $\hat{\boldsymbol{\sigma }}^{2}(a)=\sum _{i=1}^n \{ (\boldsymbol{X}_i\hat{\varepsilon }_i)(\boldsymbol{X}_i\hat{\varepsilon }_i)^T\}(a) /n$ is the sample version of ${\boldsymbol{\sigma }}^{2}(a)$. The resulting bootstrap for $M_n$ is $M_n^*=\sup _{a\in [\alpha _1,\alpha _2]} \textrm{f}_n(\hat{\Uppsi }^{*})(a)$. The relevant bootstrap consistency is established as follows (see Appendix D for the proof).

#### Corollary 2

Under the conditions of Theorem 2, for all sufficiently small δ>0, there exists $I_\delta \subset [\alpha _1,\alpha _2]$ having Lebesgue measure δ such that $\textrm{f}_n(\hat{\Uppsi }^{*})^2(a)$ converges weakly to $\boldsymbol{g}\left( \boldsymbol{E}\right) ^T \left( a\right) \boldsymbol{\sigma }^{-2}\left( a\right) \boldsymbol{g}\left( \boldsymbol{E}\right) \left( a\right)$ in $\ell ^\infty ( [\alpha _1,\alpha _2] \setminus I_\delta )$ as $n \rightarrow \infty$, given the data sequence $\{\textrm{f}_j(Y_i), \textrm{f}_j(\boldsymbol{X}_i), i=1,\ldots , j, j=1, 2, \ldots \}$, in probability.

Corollary 2 provides an asymptotic $100(1-\alpha )\%$ simultaneous confidence band for $\beta _j(\cdot )$ for essentially all $a\in [\alpha _1,\alpha _2]$:

$$ \{ (a,\tilde{\beta }_j(a)): -2 \log \textrm{f}_n(\mathcal {R}(\tilde{\beta }_j))(a) \le c^*_{EL, \alpha }, \tilde{\beta }_j \in \mathcal {D}_n\},$$

where $c^*_{EL, \alpha }$ denotes the upper α-quantile of the $M_n^*$ values obtained from B=1000 (as in Section 2.1) bootstrap samples, and $\mathcal {D}_n$ is the class of functions of the form in Eq. 1. We refer to this as the *EL band*. See Algorithm 1 for a step-by-step implementation for constructing the band. In the algorithm, $\textbf{V}$ represents the grid of points in $[\alpha _1, \alpha _2]$ where the band is evaluated. This grid is designed to numerically approximate $[\alpha _1, \alpha _2]$ and is typically denser than $\textbf{G}_n$. After getting $c^*_{EL, \alpha }$, the algorithm initiates the construction of the EL band for $a \in \textbf{G}_n$ and subsequently expands the band to include $a \in \textbf{V}$ using $ind_{b_a}$. Here, $ind_{b_a}$ provides an index for $b_a$ in Eq. 1 with respect to $\textbf{G}_n$, corresponding to the smallest element in $\textbf{G}_n$ that is no less than a given $a \in \textbf{V}$.

By a similar reasoning as in Section 7.2 of Chang and McKeague (2022b), it can be shown that if the response function is monotone in *a* and the covariate functions are constant in *a*, then the lower and upper boundaries of the EL band will respect this monotonicity. An example of a monotonic response function and constant covariate functions is given in our data analysis (see Section 4), where the response is the occupation time curve and the covariates are age and intercept terms.

The NS band we introduced in Section 2.1 also respects such monotonicity, by the fact that $\textrm{f}_n(\hat{\boldsymbol{\beta }})(a)$ is monotone in *a* and $n^{-1/2} c^*_{NS, \alpha }$ is constant in *a*. (See Figure 2 of Section 4 for an illustration of the monotonicity of both the EL and NS bands.) However, as mentioned in Section 2.1, the NS band is not an optimal band.

Note that both the EL and NS bands can be used for simultaneous testing of $H_0 :\beta _j(a) = \beta _{j0}(a)$ versus $H_1:H_0$ is not true, for some given function $\beta _{j0}(a)$. The EL/NS test rejects $H_0$ if there exists an $a \in [\alpha _1, \alpha _2]$ such that the corresponding EL/NS band fails to capture $\beta _{j0}(a)$.

**Algorithm 1:**
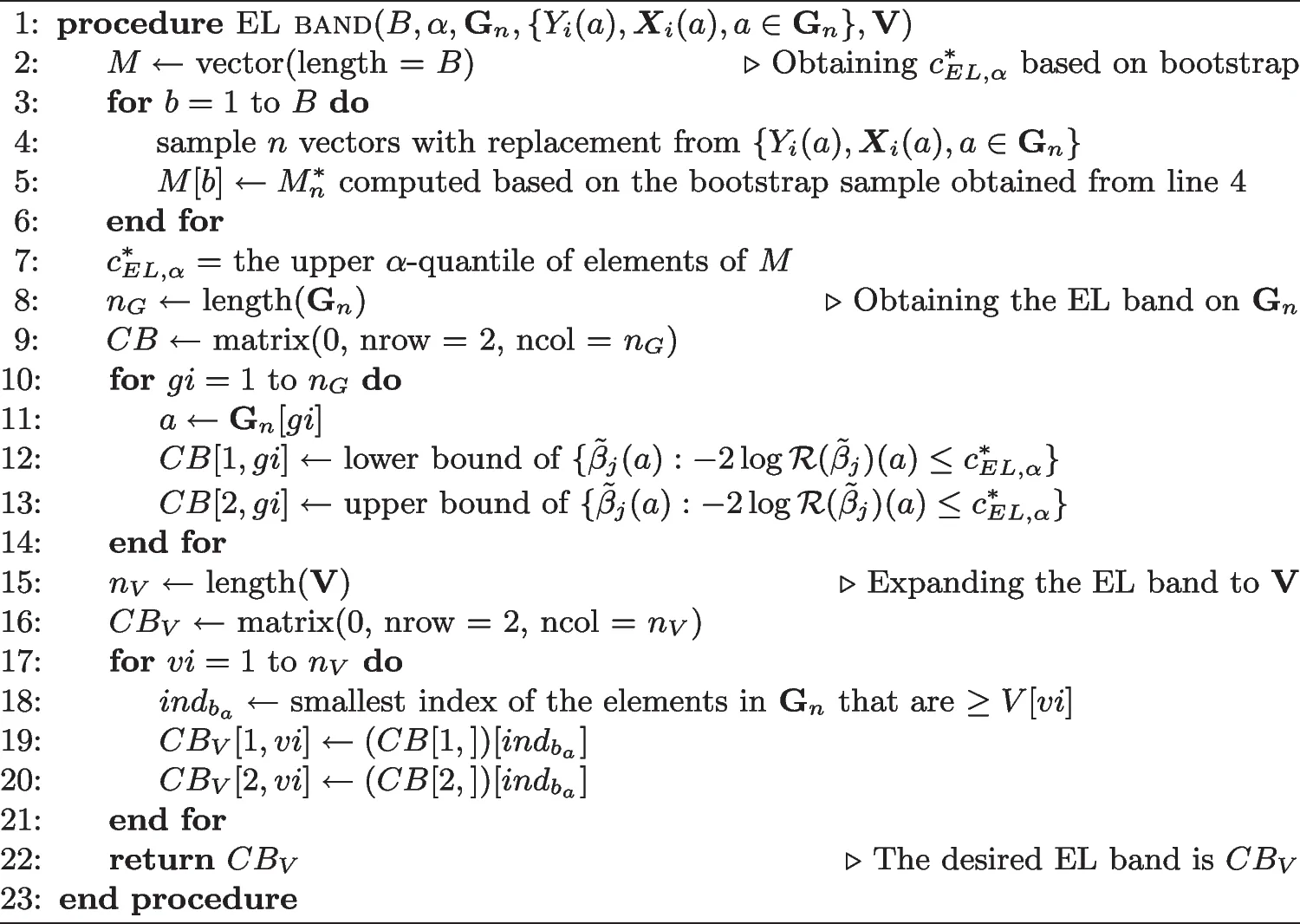
Obtaining the EL band based on $c^*_{EL, \alpha }$.

## Simulation Study

In this section, we compare the performance of the proposed simultaneous confidence band with the NS band in Section 2.1. In addition, we compare the EL-band-based test (see the last paragraph of the previous section) with a smoothing-based simultaneous test devised by Wang et al. (2018), along with a version of EL-band-based test with $c^*_{EL, \alpha }$ replaced by a critical value based on evaluating $M_n$ on each bootstrap sample.

We generate $\boldsymbol{X}(a) = (1, T(a))^T$ and $\boldsymbol{\beta }(a) = (1, \beta _2(a))^T$, where T(a)=max(J(a),0), a∈[0,1], *J*(*a*) is a zero-mean Gaussian process having a non-smooth $\textrm{Cov}\{J(a),J(b)\} =$ $10.6 I\{a< 0.25, a = b \} + 0.6 I\{a \ge 0.25, a = b \} + 1.5 I\{a, b < 0.25, a \ne b\} + 0.5 I\{a \text{ or } b \ge 0.25, a \ne b\}$, and $\beta _2(a) = \xi I\{a \ge 0.25\}$ is a non-smooth regression coefficient function with jump $\xi \ne 0$. Futher, we simulate the error process $\varepsilon (a) \sim \text{ Uniform }(-s, s)$ for some s>0 and obtain the response function

$$ Y(a) = \boldsymbol{\beta }^T(a)\boldsymbol{X}(a) + \varepsilon (a). $$

We use a regular grid $\textbf{G}_n$ of 26 points and n=50,100,200,300. The coverage is evaluated on a regular grid $\textbf{V}$ of 101 points.

**Table 1:** Simulation study of 95% simultaneous confidence bands for $\beta _2(a)$: empirical coverage (percentage) and average width (in parenthesis); 1000 Monte Carlo replications, each with 1000 bootstrap samples, jump parameter ξ=1,2, uniform location parameter s=0.05,0.1, n=50,100,200,300

*n*	bands	ξ=2		ξ=1	
		s=0.1	s=0.05	s=0.1	s=0.05
50	EL	84.7	84.5	84.6	84.8
		(0.046)	(0.023)	(0.046)	(0.023)
	NS	95.7	95.2	95.4	95.0
		(0.058)	(0.029)	(0.058)	(0.029)
100	EL	92.0	92.3	92.1	92.3
		(0.032)	(0.016)	(0.032)	(0.016)
	NS	95.7	96.4	96.4	96.4
		(0.039)	(0.020)	(0.039)	(0.020)
200	EL	94.3	94.1	94.8	94.2
		(0.022)	(0.011)	(0.022)	(0.011)
	NS	94.6	95.1	94.9	94.6
		(0.028)	(0.014)	(0.028)	(0.014)
300	EL	95.1	95.0	95.0	95.1
		(0.018)	(0.009)	(0.018)	(0.009)
	NS	95.3	95.2	95.2	95.2
		(0.022)	(0.011)	(0.022)	(0.011)

The time required to run one dataset generated from the above model with ξ=2, s=0.1, a regular grid $\textbf{G}_n$ of 3 points (for illustration only), and n=50 is around 2.5 minutes for the EL band and <1 second for the HW band. In contrast, the smoothing-based simultaneous test and the alternative EL test took roughly 20.5 minutes and 1.2 hours to run, respectively. These computations were performed on a computer with an Intel Xeon E5-2620 v4 CPU @ 2.10 GHz and 384 GB RAM. While the EL band takes longer to compute than the HW band, we believe the improved efficiency in utilizing the data justifies the additional computational time.

Empirical coverage rates and average widths of the two simultaneous confidence bands for $\beta _2(a)$ are given in Table 1, where we define the width of a band as the average width over the range of activity levels. In all cases considered, our EL band is narrower (i.e., more efficient) than the NS band. However, it exhibits anti-conservativeness in smaller samples (n=50,100), a phenomenon well-documented in the empirical likelihood literature (see, e.g., Emerson and Owen 2009). Fortunately, this anti-conservative behavior diminishes as the sample size increases to n≥200. In other words, when n=200 and 300, the empirical coverage rates of our EL band and the NS band are both close to the nominal 95% level, but NS is wider than the EL band. Since our real data example in Section 4 involves n>500, we anticipate the accuracy of our EL band in that situation. Nevertheless, for smaller samples, we acknowledge the accuracy of the NS band and recommend its usage. For moderate sample sizes, we conclude that the proposed EL confidence bands have better performance in terms of the properties mentioned above.

Empirical rejection rates of the simultaneous test for $H_0 :\beta _j(a) = 0$ versus $H_1:H_0$ is not true based on the EL- (labeled as EL in the table), alternative EL-band (labeled as EL2 in the table), and a smoothing-based simultaneous test (labeled as sm in the table) devised by Wang et al. (2018), are given in Table 2. Under $H_0$ (i.e., when ξ=0), the empirical levels based on EL and EL2 are close to the nominal level of 0.05. In contrast, the smoothing-based test is extremely inaccurate, having an empirical rejection rate of 1 under $H_0$. Under $H_1$ (i.e., when ξ>0), EL is slightly more powerful than EL2. On the other hand, the power of the smoothing-based test exceeds those of EL and EL2 tests, due to the inaccurate calibration evident in the previous results under $H_0$. Based on these results, we can see the advantage of using the proposed EL approach in non-smooth situations, and that there is no need to use the more computationally intensive EL2 approach.

**Table 2:** Simulation study of simultaneous testing of $H_0 :\beta _2(a) = 0$ versus $H_1:H_0$ is not true, at a nominal level of 5%: empirical rejection rates (percentages) and their corresponding empirical standard deviations (percentages, in parentheses); 450 Monte Carlo replications, each with 1000 bootstrap samples, jump parameter ξ=0,0.005, uniform location parameter s=0.1, n=300

ξ	tests		
	EL	EL2	sm
0	4.7	3.8	1
	(1.00)	(0.90)	(0.00)
0.005	56.7	54.7	1
	(2.34)	(2.35)	(0.00)

## Real Data Example

The occupation time data we use are based on wearable device measurements from the 2005–06 U.S. National Health and Nutrition Examination Survey (United States National Center for Health Statistics, 2006). Each raw activity curve was measured in one-minute epochs using a wearable ActiGraph device for seven consecutive days (normalized to [0,τ]=[0,1]); we only keep measurements that NHANES flagged as both “reliable” and “in calibration”.

We restrict attention to veteran subjects of ages ranged from 17 to 85 (n=571); systematically selected occupation time curves are displayed in the left panel of Figure 2. We consider the maximal range of activity levels that has been categorized as sedentary in existing physical activity literature (Gorman et al., 2014), namely <500 counts/minute. The occupation time curves are restricted to the interval $[\alpha _1, \alpha _2] = [0, 499]$ and discretized at each intensity count (i.e., integers). There are no missing data among these veterans.

**Fig. 1:**
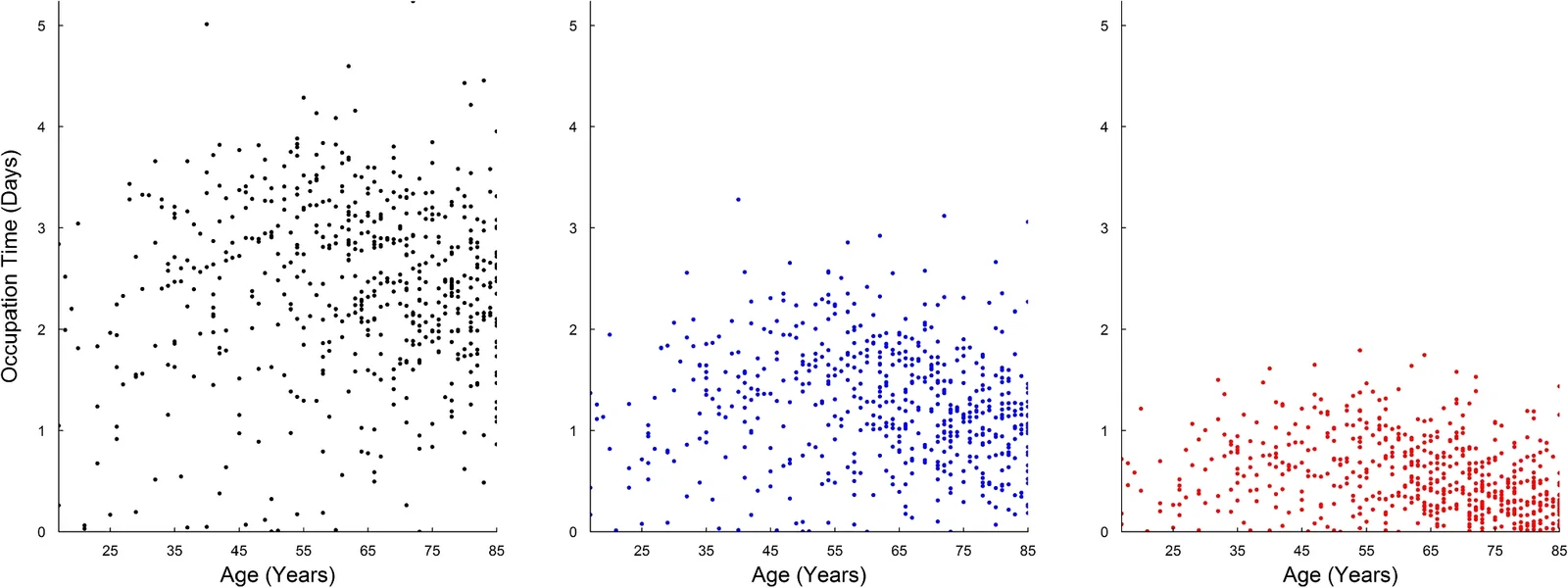
Scatter plots of occupation time (in days over one week) versus age (in years) over a=0 (left panel), 114 (middle panel) and 499 (right panel) counts/minute

We are interested in the effect of age on the mean occupation time curve among the veteran subjects. From the scatter plots in Figure 1, it seems that there is an age effect in higher activity levels. To make more rigorous inference, we construct a confidence band for the regression coefficient function of age in the model

$$ \text {E}(\text {occupation time}(a) | \text {age}) = \beta _0(a) + \beta _1(a) \times \text {age} $$

for all $a \in [\alpha _1, \alpha _2]$. Note that we center age during the implementation, although it does not affect the desired confidence band.

**Fig. 2:**
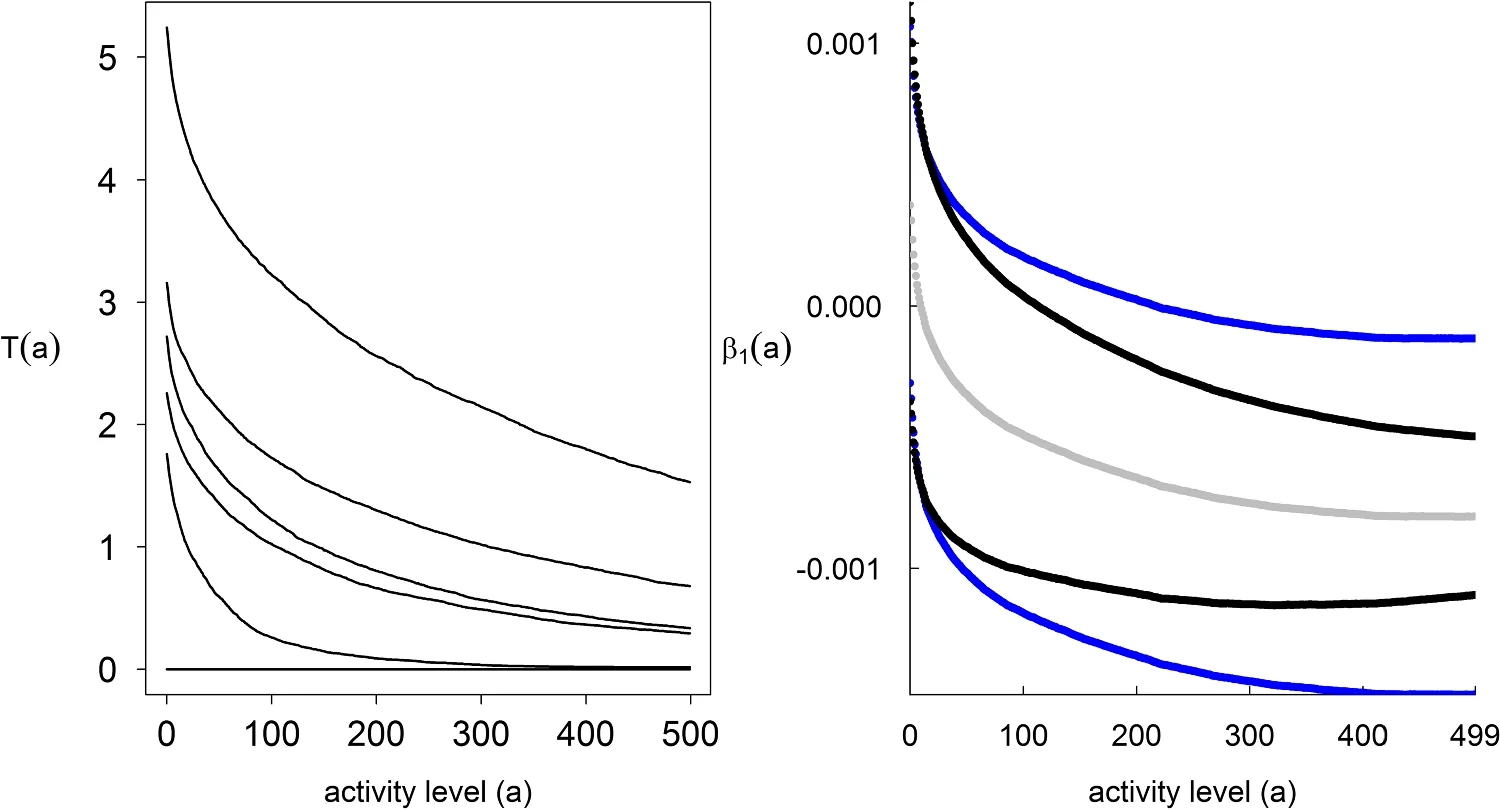
Left panel: occupation time (in days over one week) from systematically selected (the smallest and largest observations, and every twentieth percentile at a=0) veterans of all ages, based on the NHANES physical activity data. Right panel: EL (black) and NS (blue) 95% simultaneous confidence bands for the regression coefficient function of age (estimate in gray) among veterans

Comparing the confidence bands based on EL and NS (see the right panel of Figure 2), we can see that the EL band is narrower than NS, which is consistent with the simulation results in Table 1. Consequently, the EL band leads to more significant results than the NS band. More specifically, the EL band for the regression coefficient function of age includes 0 for lower activity levels, but decreases below 0 for activity levels ≥114 counts/minute. This means there is no significant age effect for the lower activity levels; however, for those higher activity levels, age is negatively associated with mean occupation time, with activity level 499 counts/minute being the activity level of greatest age impact. In particular, an increase in ten years of age will result in a (50, 111)-minute decrease in the mean occupation time over one week above activity level 499 counts/minute (i.e., the minimal quantification of sedentary behavior in the literature). Note that these results are consistent with the finding illustrated in the middle panel of Figure 5 in Chang and McKeague (2022a), where there is a larger positive difference of mean occupation time curves at higher activity levels when comparing younger versus older age groups among veterans, although now we consider a larger age range.

## Discussion

In this paper we develop a CFL regression method that allows left- (or right-) discontinuities in the regression coefficient function of interest and the relevant functional means and covariances under a dense non-random design. Although the consideration of discontinuities is motivated by occupation time data derived from wearable devices, our method applies to general functional data that satisfy the conditions in our theory. Taking advantage of optimality properties and the ability to handle the monotonicity of these occupation time curves, our EL approach is used to construct a simultaneous confidence band for one regression coefficient function of interest while adjusting for other covariates. We have shown via simulations that the proposed confidence band maintains accuracy in moderate sample sizes when there is a discontinuity in the regression coefficient function of interest and the means and covariances of the covariate and response functions, while being narrower than an alternative Wald-type approach. In addition, a simultaneous test based on the proposed confidence band is shown to substantially outperform an existing test based on smoothing. We applied the proposed procedures to wearable device data from the 2005–06 NHANES study, obtaining narrower confidence bands than the Wald-type band; we also obtain more significant results in the simultaneous test.

Although we can accommodate discontinuities, our approach differs from that used in change point problems. The reasons are as follows. First, we are not interested in estimating/selecting the change points (see, e.g., Bai 2010). Our goal is to contruct a confidence band for the coefficient function of interest. Even if an attempt is made to modify a change point method to do this, it would be necessary to treat those change points as plug-in estimates. Such an approach would lead to a much more complicated theory. Second, our assumptions regarding the functional response and predictors are different from certain assumptions such as parametric models (see, e.g., Bélisle et al. 1998), smooth conditional covariances (see, e.g., Zhu et al. 2014), or stationary (see, e.g., Xi and Pang 2021) used in the change point literature. The key for us is to utilize the right- (or left-) continuity of the coefficient function of interest and the data, which is enough for the above assumptions to be relaxed. Last but not least, our work addresses an important feature of functional data analysis that treats the observed trajectories as discretized versions of an underlying trajectory and is able to make inference about the complete trajectory. In contrast, the change point literature is not known to do that.

## Data Availability

Publicly available.

## Appendix A Proof of Theorem 1

For this proof and all other proofs in the supplement, $o_p$, *o*, $O_p$ and *O* refer to *uniform* bounds over $a \in [\alpha _1,\alpha _2]\setminus I_{\delta }$.

### A.1 The First Part

Since $\sqrt{n} \left\{ \textrm{f}_{n}(\hat{\boldsymbol{\beta }}) - \textrm{f}_{n}(\boldsymbol{\beta }) \right\} (a)$ can be written as

A1 $$\begin{aligned}&\left\{ \sum _{i = 1}^n \textrm{f}_{n}\left( \boldsymbol{X}_i \boldsymbol{X}_i^T\right) / n\right\} ^{-1}\left( a\right) \times \nonumber \\&\sqrt{n} \left\{ \sum _{i = 1}^n \textrm{f}_{n}\left( \boldsymbol{X}_i Y_i\right) / n - \sum _{i = 1}^n \textrm{f}_{n}\left( \boldsymbol{X}_i \boldsymbol{X}_i^T\boldsymbol{\beta }\right) / n \right\} \left( a\right) \end{aligned}$$

by definition of $\hat{\boldsymbol{\beta }}(a)$ in Section 2.1, we begin by studying the asymptotics of the components in Eq. A1. More specifically, we derive the limits of $\sum _{i = 1}^n \textrm{f}_{n}\left( \boldsymbol{X}_iX_{ij}\right) \left( a\right) / n$ and $\textrm{f}_{n}({\boldsymbol{E}}_n)(a)$ in $(\ell ^\infty ( [\alpha _1,\alpha _2]\setminus I_{\delta }))^{p}$ as $n \rightarrow \infty$, where $\varepsilon _i(a) = (Y_i-\boldsymbol{X}_i^T\boldsymbol{\beta })(a)$, $I_{\delta }$ can be constructed as $\bigcup _{\ell =1}^{J}(c_{\ell }-\delta /J, c_{\ell })$, $c_{\ell }$ for $\ell =1, \ldots , J < \infty$ are the (finitely many) discontinuities of $E(X_j\varepsilon )(\cdot )$ and $E(X_jX_{\ell })(\cdot )$, $X_j(a)$ and $X_{ij}(a)$ is the *j*-th element of $\boldsymbol{X}(a)$ and $\boldsymbol{X}_i(a)$, respectively ($j, \ell = 1, \ldots , p$), and δ can be any small positive number that is less than the minimal distance between any two $c_{\ell }$’s and between any $c_{\ell }$ and $\alpha _1$ or $\alpha _2$, divided by 2. Then we use the continuous mapping theorem to get the desired limiting process of $\sqrt{n} \left\{ \textrm{f}_{n}(\hat{\boldsymbol{\beta }}) - \textrm{f}_{n}(\boldsymbol{\beta }) \right\} (a)$.

To obtain the limiting process of $\textrm{f}_{n}({\boldsymbol{E}}_n)(a)$, we begin with finding the limiting distributions of $\sum _{i = 1}^n \textrm{f}_{n}(X_{i, j}\varepsilon _i)(a) / \sqrt{n}$. By checking the conditions of a changing-class Donsker Theorem (Kosorok, 2008b; Kosorok, 2008a) in a similar way as in Supplement Section 1.1 of Chang and McKeague (2022b), we can show that for each j=1,…,p, $\sum _{i = 1}^n \textrm{f}_{n}(X_{i, j}\varepsilon _i)(a) / \sqrt{n}$ converges in distribution in $\ell ^\infty ( [\alpha _1,\alpha _2]\setminus I_{\delta })$ to a tight Gaussian process with zero mean and covariance function $E\left\{ K(a, b) X_j\left( a\right) X_j\left( b\right) \right\}$. Note that in the process of checking those conditions for the changing classes Donsker Theorem, we make use of the conditions (see the statement of Theorem 1) that the sample paths of $X_j(a)$ and ε(a) are right-continuous, of bounded variation, bounded by some constant τ, and $E(\boldsymbol{X}\varepsilon )(\cdot )$ has at most finitely many jump discontinuities.

Then we utilize an extension of the characterization of weak convergence as asymptotic tightness plus convergence of marginals to multidimensional bounded stochastic processes (see, e.g., van der Vaart and Wellner 1996, Theorem 1.5.4). Specifically, we show the convergence of all finite-dimensional marginals, and then asymptotic tightness. To show convergence of all finite-dimensional marginals, fix any $a_1, \ldots , a_k \in ([\alpha _1,\alpha _2]\setminus I_{\delta })$. By the multivariate central limit theorem, $[\textrm{f}_{n}({\boldsymbol{E}}_n^T)(a_1), \textrm{f}_{n}({\boldsymbol{E}}_n^T)(a_2), \ldots ,\textrm{f}_{n}({\boldsymbol{E}}_n^T)(a_k)]^T$ converges weakly to a mean zero multivariate normal distribution. To prove the asymptotic tightness of $\textrm{f}_{n}({\boldsymbol{E}}_n)(a)$, it suffices to show that each coordinate is asymptotically tight (see, e.g., van der Vaart and Wellner 1996, Lemma 1.4.3), which follows by the fact that the limit of each coordinate, namely the aforementioned limiting process of $\sum _{i = 1}^n \textrm{f}_{n}(X_{i, j}\varepsilon _i)(a) / \sqrt{n}$ for each j=1,…,p, is tight (see, e.g., van der Vaart and Wellner 1996, Lemma 1.3.8). These results also imply the existence of a tight limiting process of $\textrm{f}_{n}({\boldsymbol{E}}_n)(a)$. Further, this limiting process has a marginal distribution being the above mean zero multivariate normal distribution. This implies the limiting process of $\textrm{f}_{n}({\boldsymbol{E}}_n)(a)$ is a tight multivariate Gaussian process ${\boldsymbol{E}}(a)$ with zero mean and covariance structure $E\left\{ K(a, b) \boldsymbol{X}\left( a\right) \boldsymbol{X}^T\left( b\right) \right\}$.

As for the limit of $\sum _{i = 1}^n \textrm{f}_{n}\left( \boldsymbol{X}_iX_{ij}\right) \left( a\right) / n$, it suffcies to show

A2 $$\begin{aligned} \sum _{i = 1}^n \textrm{f}_{n}\left( X_{ij}X_{i\ell }\right) \left( a\right) / n \rightarrow E\left( X_{j}X_{\ell }\right) \left( a\right) \end{aligned}$$

a.s. in $\ell ^\infty ( [\alpha _1,\alpha _2]\setminus I_{\delta })$ as $n \rightarrow \infty$, for all $j, \ell = 1, \ldots , p$. For each $j, \ell = 1, \ldots , p$, we first obtain

A3 $$\begin{aligned} \sup _{a \in [\alpha _1,\alpha _2]} \left| \sum _{i = 1}^n \textrm{f}_{n}\left( X_{ij}X_{i\ell }\right) \left( a\right) / n - \textrm{f}_{n}\left( E\left( X_{j}X_{\ell }\right) \right) \left( a\right) \right| \rightarrow 0 \end{aligned}$$

a.s. as $n \rightarrow \infty$, by the fact that the LHS of Eq. A3 is no greater than $\sup _{a \in [\alpha _1,\alpha _2]} \left| \sum _{i = 1}^n \left( X_{ij}X_{i\ell }\right) \left( a\right) / n - E\left( X_{j}X_{\ell }\right) \left( a\right) \right| \rightarrow 0$ a.s. The last convergence to 0 is by the Glivenko–Cantelli theorem because the sample path of $X_{j}$ (and hence $X_j X_{\ell }$) has bounded variation and is bounded by some constant (see, e.g., Chang and McKeague 2022b, the second paragraph on page 8). Secondly, it can be shown that

A4 $$\begin{aligned} \sup _{a \in [\alpha _1,\alpha _2]\setminus I_{\delta }} \left| \textrm{f}_{n}\left( E\left( X_{j}X_{\ell }\right) \right) \left( a\right) - E\left( X_{j}X_{\ell }\right) \left( a\right) \right| \rightarrow 0 \end{aligned}$$

a.s. as $n \rightarrow \infty$, by a similar reasoning as in Section 1.2 of Chang and McKeague (2022b) and the fact that $E\left( X_{j}X_{\ell }\right) \left( \cdot \right)$ is right-continuous, of bounded variation, with at most finitely many jump discontinuities. Then by Eqs. A3, A4 and the triangle inequality, we get the desired result in Eq. A2.

The result in the previous paragraph implies

A5 $$\begin{aligned} \textrm{f}_{n}\left( \sum _{i = 1}^n \text{ vec }\left( \boldsymbol{X}_{i} \boldsymbol{X}_{i}^T\right) / n\right) \left( a\right) \rightarrow \text{ vec }\left\{ E\left( \boldsymbol{X} \boldsymbol{X}^T \right) \right\} \left( a\right) \end{aligned}$$

a.s. in $(\ell ^\infty ( [\alpha _1,\alpha _2]\setminus I_{\delta }))^{p^2}$ as $n \rightarrow \infty$, where $\text{ vec }(\cdot )$ denotes vectorization. This and the asymptotic result of $\textrm{f}_{n}({\boldsymbol{E}}_n)(a)$ above imply the desired result in the first display of Theorem 1, by the continuous mapping theorem, $\inf _{a \in [\alpha _1,\alpha _2]} \det \{E(\boldsymbol{X} \boldsymbol{X}^T)\}(a) > 0$, and the fact that the sample paths of $X_j(a)$ is bounded by some constant τ.

### A.2 The Second Part

The result follows if we can show $\sqrt{n} \{\textrm{f}_n(\boldsymbol{\beta })(a) - \boldsymbol{\beta }(a)\} = o(1)$ as $n \rightarrow \infty$. It suffices to show $\sqrt{n} \{\textrm{f}_n(\beta _j)(a) - \beta _j(a)\} = o(1)$ as $n \rightarrow \infty$ for each j=1,…,p, which follows by the same reasoning as in Section 1.2 of Chang and McKeague (2022b) and the additional conditions in the second part of Theorem 1.

## Appendix B Proof of Corollary 1

To show bootstrap consistency of $$f_{n,a}(U_n^{*})$$, we write it as $$U_n^*(a) = \left\{ \sum _{i = 1}^n (\boldsymbol{X}_i \boldsymbol{X}_i^T)/ n\right\} ^{-1}\left( a\right) \sum _{i=1}^{n} \{(W_{ni} - 1)\boldsymbol{X}_i(a) \hat{\varepsilon }_i(a)\} /\sqrt{n}$$, where $$W_{ni}$$ is the number of times that $$f_n(\boldsymbol{X}_i \hat{\varepsilon }_i)(a)$$ is redrawn from $$\{\textrm{f}_n(\boldsymbol{X}_1\hat{\varepsilon }_1)(a), \!\ldots \! , \textrm{f}_n(\boldsymbol{X}_n\hat{\varepsilon }_n\!) (a), a \in [\alpha _1, \alpha _2]\}$$. To eliminate the dependence among $$\{\hat{\varepsilon }_i(a)\}_{i=1}^n$$, we introduce $$U_n^{**}(a) = \left\{ \sum _{i = 1}^n (\boldsymbol{X}_i \boldsymbol{X}_i^T)/ n\right\} ^{-1}\left( a\right) \sum _{i=1}^{n} \{(W_{ni} - 1)\boldsymbol{X}_i(a) \varepsilon _i(a)\} /\sqrt{n}$$. We first show the conditional asymptotic equivalence of $$f_{n,a}(U_n^{*})$$ and $$f_{n,a}(U_n^{**})$$. Secondly, we show the bootstrap consistency of $$f_{n,a}(U_n^{**})$$. The aforementioned bootstrap consistency results are provided conditional on $$\{Y_1(a), \boldsymbol{X}_1(a)\}, \{Y_2(a), \boldsymbol{X}_2(a)\}, \ldots $$, in probability. Then the extension to conditioning on the discretized data follows by a similar reasoning in the last paragraph of Section 3.1 of Chang and McKeague (2022b).

We show the conditional asymptotic equivalence of $$\textrm{f}_n(U_n^*)(a)$$ and $$\textrm{f}_n(U_n^{**})(a)$$ as follows. It amounts to showing that

B6 $$\begin{aligned}&U_n^{**}(a) - U_n^*(a) = \left\{ \sum _{i = 1}^n \left( \boldsymbol{X}_i \boldsymbol{X}_i^T\right) / n\right\} ^{-1} \left( a\right) \times \nonumber \\&\frac{1}{\sqrt{n}}\sum _{i=1}^{n} \left\{ (W_{ni} - 1)\boldsymbol{X}_i^T(a)\right\} \left( \hat{\boldsymbol{\beta }} - \boldsymbol{\beta }\right) (a) = o_p(1) \end{aligned}$$

conditional on $\{Y_1(a), \boldsymbol{X}_1(a)\}, \{Y_2(a), \boldsymbol{X}_2(a)\}, \ldots$, in probability. Each dimension j=1,…,p of the middle term $\sum _{i=1}^{n} \left\{ (W_{ni} - 1)X_{ij}(a)\right\} / \sqrt{n}$ after the first equality converges weakly to the limiting process of $\sum _{i=1}^{n} \left\{ X_{ij}(a) - E\right. \left. (X_{ij})(a)\right\} / \sqrt{n}$ in $\ell ^\infty ([\alpha _1,\alpha _2])$ as $n \rightarrow \infty$, given $\{Y_1(a), \boldsymbol{X}_1(a)\}, \{Y_2(a), \boldsymbol{X}_2 (a)\}, \ldots$, in probability, by Corollary S.1 of Chang and McKeague (2022b). The first term $\left\{ \sum _{i = 1}^n (\boldsymbol{X}_i \boldsymbol{X}_i^T)/ n\right\} ^{-1}\left( a\right)$ after the first equality is $O_p(1)$ conditional on $\{Y_1(a), \boldsymbol{X}_1(a)\}, \{Y_2(a), \boldsymbol{X}_2(a)\}, \ldots$, in probability, by Markov’s inequality and a similar reasoning as in the last two paragraphs of Appendix A.1.

The last term $(\hat{\boldsymbol{\beta }} - \boldsymbol{\beta })(a)$ of Eq. B6 after the first equality can be written as $\left\{ \sum _{i = 1}^n (\boldsymbol{X}_i \boldsymbol{X}_i^T)/ n\right\} ^{-1}\left( a\right) \left\{ \sum _{i = 1}^n \left( \boldsymbol{X}_i \varepsilon _i\right) / n \right\} \left( a\right)$, which can be shown to be $o_p(1)$ conditional on $\{Y_1(a), \boldsymbol{X}_1(a)\}, \{Y_2(a), \boldsymbol{X}_2(a)\}, \ldots$, in probability. This is true due to the last result in the previous paragraph, Markov’s inequality, and the Glivenko–Cantelli theorem because the sample paths of $\boldsymbol{X}(a) \varepsilon (a)$ have bounded variation and is uniformly bounded (see, e.g., Chang and McKeague 2022b, the second paragraph on page 8). Combining the results from the three terms of Eq. B6 after the first equality, and a routine extension of the conditional Slutsky’s lemma (Cheng 2015, Appendix A.2, (i), (ii)) to the case of random elements of a metric space, leads to the $o_p(1)$ in Eq. B6 conditional on $\{Y_1(a), \boldsymbol{X}_1(a)\}, \{Y_2(a), \boldsymbol{X}_2(a)\}, \ldots$, in probability.

To show the bootstrap consistency of $$\textrm{f}_{n,a}(U_n^{**})$$, we can use Poissonization and a changing classes bootstrap central limit theorem in a similar way as in Section 3.1 of Chang and McKeague (2022b). More specifically, a partly Poissonized version of $$\textrm{f}_{n,a}(U_n^{**})$$ is defined as $$\textrm{f}_{n,a}(U_{n,N_n}^{**})$$, where $$U_{n,N_n}^{**}(a) = \left\{ \sum _{i = 1}^n (\boldsymbol{X}_i \boldsymbol{X}_i^T)/ n\right\} ^{-1}(a) \sum _{i=1}^{n} \{(W_{N_n,i}-1) \boldsymbol{X}_i(a) \varepsilon _i(a)\} /\sqrt{n}$$ and $$N_n$$ is a Poisson random variable with mean *n* and is independent of the original sample. We can obtain the conditional asymptotic equivalence of $$\textrm{f}_{n,a}(U_n^{**})$$ and $$\textrm{f}_{n,a}(U_{n,N_n}^{**})$$ by the the Glivenko–Cantelli result for $$\left\{ \sum _{i = 1}^n \left( \boldsymbol{X}_i \varepsilon _i\right) / n \right\} \left( a\right) $$ in the previous paragraph. Then we show the bootsrap consistency of $$\textrm{f}_{n,a}(U_{n,N_n}^{**})$$ by the fact that $$ \sum _{i=1}^{n}\{ (\frac{W_{N_n,i}}{N_n/n}-1) \textrm{f}_{n,a}(\boldsymbol{X}_{i}\varepsilon _{i})\}/\sqrt{n} $$ satisfies a changing classes bootstrap central limit theorem (Kosorok 2008b, Theorem 11.23), and by a routine extension of this theorem to the case of multidimensional empirical process. Finally, by a routine extension of the conditional Slutsky’s lemma (Cheng 2015, Appendix A.2, (i)) to the case of random elements of a metric space, and the conditional asymptotic equivalence of $$\textrm{f}_{n,a}(U_n^{**})$$ and $$\textrm{f}_{n,a}(U_{n,N_n}^{**})$$, we have the bootstrap consistency of $$\textrm{f}_{n,a}(U_n^{**})$$.

## Appendix C Proof of Theorem 2

In this section, $I_\delta$ can be constructed as $\bigcup _{\ell =1}^Q(d_{\ell }-\delta /Q, d_{\ell })$, $d_{\ell }$ for $\ell =1, \ldots , Q < \infty$ are the (finitely many) discontinuities of $E(X_j\varepsilon )(\cdot )$, $E(X_jX_{\ell })(\cdot )$, and $\boldsymbol{\sigma }^2(\cdot )$, and δ can be any small positive number that is less than the minimal distance between any two $d_{\ell }$’s and between any $d_{\ell }$ and $\alpha _1$ or $\alpha _2$, divided by 2.

To show the weak convergence of $-2\log f_n(\mathcal {R}(\beta _j))(\cdot )$ as $n \rightarrow \infty$, first we use Lagrange’s method to get

C7 $$\begin{aligned} -2\log f_n\left( \mathcal {R}(\beta _j)\right) (a)=2\sum _{i=1}^n \log \{1+f_n\left( \boldsymbol{\lambda }\right) \left( a\right) f_n\left( \boldsymbol{Z}_{i}\right) \left( a\right) \}, \end{aligned}$$

where $\boldsymbol{\lambda }(a)$ satisfies the estimating equation $\sum _{i=1}^n p_i(a) \boldsymbol{Z}_{i}(a)=0$, $\boldsymbol{Z}_{i}(a)=\{\boldsymbol{X}_i(Y_i - \sum _{\ell \ne j}\hat{\beta }_{\ell } X_{i\ell } - \beta _jX_{ij})\}(a)$, and $p_i(a)= [n \{1+\boldsymbol{\lambda }(a) \boldsymbol{Z}_{i}(a)\} ]^{-1}$.

Since Eq. C7 has the same form as the EL statistic in Supplement Section 4 of Chang and McKeague (2022b), we can use similar reasoning to show $f_n(\boldsymbol{\lambda })(a)=O_p(1/\sqrt{n})$. We also need the following additional large sample results: $f_n(\overline{\boldsymbol{Z}})(a)= O_p(1/\sqrt{n})$,

C8 $$\begin{aligned} f_n\left( \overline{\boldsymbol{\sigma }}^2\right) \left( a\right) = \boldsymbol{\sigma }^2\left( a\right) + o_p\left( 1\right) \end{aligned}$$

and $\max _{i = 1, \ldots , n}$ $\left\| f_n(\boldsymbol{Z}_i)(a)\right\| = O_p(1)$, where $\overline{\boldsymbol{Z}}(a) = \sum _{i=1}^n {\boldsymbol{Z}}_{i}(a) / n$, $\overline{\boldsymbol{\sigma }}^2(a) = \sum _{i=1}^n {\boldsymbol{Z}}_{i}(a){\boldsymbol{Z}}^T_{i}(a) / n$, and recall $\boldsymbol{\sigma }^2(a)= E\left\{ K(a, b) \boldsymbol{X}\left( a\right) \boldsymbol{X}^T\left( b\right) \right\}$ is defined in Section 2.2. To show $f_n(\overline{\boldsymbol{Z}})(a)= O_p(1/\sqrt{n})$, we decompose $\sqrt{n} f_n(\overline{\boldsymbol{Z}})(a)$ as

C9 $$\begin{aligned} \textrm{f}_{n}({\boldsymbol{E}}_n)\left( a\right) - \frac{1}{n} \sum _{i = 1}^n f_n\left( {\boldsymbol{X}}_{i}\right) \sum _{\ell \ne j} f_n\left( X_{i\ell }\right) \sqrt{n} \left\{ f_n\left( \hat{\beta }_{\ell }\right) - f_n\left( \beta _{\ell }\right) \right\} \left( a\right) . \end{aligned}$$

The first term in Eq. C9 has been shown to be $O_p(1)$ in Appendix A.1. The second term in Eq. C9 is $O_p(1)$ as well, because $\sqrt{n} \left\{ f_n(\hat{\beta }_{\ell }) - f_n(\beta _{\ell }) \right\} (a) = O_p(1)$ by the first part of Theorem 1, and $\sum _{i = 1}^n f_n({\boldsymbol{X}}_{i} X_{i\ell }) / n = O(1)$ a.s. by Eq. A5. Therefore, we have the desired $\sqrt{n} f_n(\overline{\boldsymbol{Z}})(a)= O_p(1)$. To show Eq. C8, note that by Eq. C9 and the above $O_p(1)$ result for its second term,

$$ f_n\left( \overline{\boldsymbol{\sigma }}^2\right) \left( a\right) = \frac{1}{n} \sum _{i=1}^n \left\{ f_n\left( {\boldsymbol{X}}_{i} \varepsilon _{i}\right) \left( a\right) + O_p\left( \frac{1}{\sqrt{n}}\right) \right\} \left\{ f_n\left( {\boldsymbol{X}}^T_{i} \varepsilon _{i}\right) \left( a\right) + O_p\left( \frac{1}{\sqrt{n}}\right) \right\} . $$

This impiles $f_n(\overline{\boldsymbol{\sigma }}^2)(a) = f_n(\boldsymbol{\sigma }^2)(a) + o_p(1)$. It remains to show that $f_n(\boldsymbol{\sigma }^2)(a) = \boldsymbol{\sigma }^2(a) + o(1)$, which is true by a similar reasoning in the last paragraph of Supplement Section 4 in Chang and McKeague (2022b) and the condition that $\boldsymbol{\sigma }^2(\cdot )$ has at most finitely many jump discontinuities. To show $\max _{i = 1, \ldots , n}$ $\left\| f_n(\boldsymbol{Z}_i)(a)\right\| = O(1)$ a.s., note that

$$\begin{aligned}&\sup _{a \in [\alpha _1,\alpha _2]\setminus I_{\delta }} \max _{i = 1, \ldots , n}\left\| f_n\left( \boldsymbol{Z}_i\right) \left( a\right) \right\| \\ \le&\sup _{a \in [\alpha _1,\alpha _2]\setminus I_{\delta }} \max _{i = 1, \ldots , n}\sqrt{\left\{ \sum _{j=1}^p f_n\left( {X}_{ij} \varepsilon _{i}\right) \left( a\right) + O_p\left( \frac{1}{\sqrt{n}}\right) \right\} ^2} \\ \le&\sup _{a \in [\alpha _1,\alpha _2]\setminus I_{\delta }} \max _{i = 1, \ldots , n}\sqrt{\sum _{j=1}^p \left\{ f_n\left( {X}_{ij} \varepsilon _{i}\right) \right\} ^2\left( a\right) + O_p\left( \frac{1}{\sqrt{n}}\right) } \\ \le&\sqrt{\sum _{j=1}^p \left\{ \sup _{a \in [\alpha _1,\alpha _2]\setminus I_{\delta }} \max _{i = 1, \ldots , n}\left| f_n\left( {X}_{ij} \varepsilon _{i}\right) \right| \left( a\right) \right\} ^2 + O_p\left( \frac{1}{\sqrt{n}}\right) }, \end{aligned}$$

where the first inequality is due to Eq. C9 and the aforementioned $O_p(1)$ result for its second term. In the last line of the above display, $\max _{i = 1, \ldots , n}\left| f_n\left( {X}_{ij} \varepsilon _{i}\right) \right| \left( a\right) = O(1)$ a.s. for each j=1,…,p because the sample paths of $X_j(a)$ and ε(a) are bounded by some constant. Therefore, we have the desired result $\max _{i = 1, \ldots , n}$ $\left\| f_n(\boldsymbol{Z}_i)(a)\right\| = O_p(1)$.

Following a similar argument in the third paragraph of page 11 in Chang and McKeague (2022b), based on the asymptotic order $f_n(\boldsymbol{\lambda })(a)=O_p(1/\sqrt{n})$, we apply Taylor’s theorem and get

C10 $$\begin{aligned} -2\log f_n\left( \mathcal {R}(\beta _j)\right) \left( a\right) = 2 n f_n(\boldsymbol{\lambda }^T\overline{\boldsymbol{Z}})\left( a\right) - n f_n\left( \boldsymbol{\lambda }^T \overline{\boldsymbol{\sigma }}^{2}\boldsymbol{\lambda }\right) \left( a\right) + o_p\left( 1\right) . \end{aligned}$$

Similarly, we expand $\sum _{i=1}^n f_n\left( p_i\right) (a) f_n\left( \boldsymbol{Z}_{i}\right) (a)$ around 0 as a function of $f_n(\boldsymbol{\lambda }^T\boldsymbol{Z}_i)(a)$ and get

C11 $$\begin{aligned} f_n\left( \boldsymbol{\lambda }\right) \left( a\right) =f_n\left( \overline{\boldsymbol{\sigma }}^{-2}\overline{\boldsymbol{Z}}\right) \left( a\right) +o_p\left( n^{-1/2}\right) \end{aligned}$$

and

C12 $$\begin{aligned} f_n\left( \boldsymbol{\lambda }^T \overline{\boldsymbol{Z}}\right) \left( a\right) =f_n\left( \boldsymbol{\lambda }^T \overline{\boldsymbol{\sigma }}^2 \boldsymbol{\lambda }\right) \left( a\right) + o_p\left( n^{-1}\right) . \end{aligned}$$

Substituting Eqs. C12 into C10 gives $-2\log f_n\left( \mathcal {R}(\beta _j)\right) (a)\!=\!nf_n\left( \boldsymbol{\lambda }^T\overline{\boldsymbol{Z}}\right) (a)\!+o_p(1)$. This and Eq. C11 imply

C13 $$\begin{aligned} -2\log f_n\left( \mathcal {R}(\beta _j)\right) \left( a\right) = n f_n\left( \overline{\boldsymbol{Z}}^T \overline{\boldsymbol{\sigma }}^{-2} \overline{\boldsymbol{Z}}\right) \left( a\right) + o_p\left( 1\right) . \end{aligned}$$

Since the asymptotic behavior of $f_n\left( \overline{\boldsymbol{\sigma }}^{2}\right)$ has already been characterized in Eq. C8, it remains to obtain the weak limit of the process $\sqrt{n} f_n\left( \overline{\boldsymbol{Z}}\right) (a)$. To this end, we have

C14 $$\begin{aligned}&\sqrt{n} f_n\left( \overline{\boldsymbol{Z}}\right) \left( a\right) = \textrm{f}_{n}({\boldsymbol{E}}_n)\left( a\right) \nonumber \\ -&\frac{1}{n} \sum _{i=1}^n f_n\left( {\boldsymbol{X}}_{i} {\boldsymbol{X}}^T_{i(-j)}\right) \left( a\right) \sqrt{n} \left\{ \textrm{f}_{n}(\hat{\boldsymbol{\beta }}) - \textrm{f}_{n}(\boldsymbol{\beta }) \right\} _{(-j)}\left( a\right) = \textrm{f}_{n}({\boldsymbol{E}}_n)\left( a\right) \nonumber \\ -&\left\{ E\left( {\boldsymbol{X}} {\boldsymbol{X}}^T_{(-j)}\right) \left( a\right) + o_p(1)\right\} \left[ \left\{ E\left( \boldsymbol{X} \boldsymbol{X}^T\right) \right\} ^{-1}_{(-j)}\left( a\right) + o_p(1)\right] \textrm{f}_{n}({\boldsymbol{E}}_n)\left( a\right) \nonumber \\ =&\textrm{f}_{n}({\boldsymbol{E}}_n)\left( a\right) - E\left( {\boldsymbol{X}} {\boldsymbol{X}}^T_{(-j)}\right) \left( a\right) \left\{ E\left( \boldsymbol{X} \boldsymbol{X}^T\right) \right\} ^{-1}_{(-j)}\left( a\right) \textrm{f}_{n}({\boldsymbol{E}}_n)\left( a\right) + o_p\left( 1\right) \nonumber \\ =&\boldsymbol{g}\left( \textrm{f}_{n}({\boldsymbol{E}}_n)\right) \left( a\right) + o_p\left( 1\right) , \end{aligned}$$

where recall from Section 2.2 that we use ${\boldsymbol{v}}_{(-j)}(a)$ to denote the vector/matrix of processes indexed by *a* after removing the *j*-th element/row of some vector/matrix of processes ${\boldsymbol{v}}(a)$, the second equality is due to Eq. A5, the continuous mapping theorem, $\inf _{a \in [\alpha _1,\alpha _2]} \det \{E(\boldsymbol{X} \boldsymbol{X}^T)\}(a) > 0$, and the fact that the sample paths of $X_j(a)$ is bounded by some constant τ, and the third equality is due to $\textrm{f}_{n}({\boldsymbol{E}}_n)(a)=O_p(1)$ by Appendix A.1, and recall from Section 2.2 that

$$ \boldsymbol{g}\left( \boldsymbol{e}\right) \left( a\right) = \boldsymbol{e}\left( a\right) - E\left( {\boldsymbol{X}} {\boldsymbol{X}}^T_{(-j)}\right) \left( a\right) \left\{ E\left( \boldsymbol{X} \boldsymbol{X}^T\right) \right\} ^{-1}_{(-j)}\left( a\right) \boldsymbol{e}\left( a\right) $$

for any $\boldsymbol{e} \in (\ell ^\infty ( [\alpha _1,\alpha _2]\setminus I_{\delta }))^{p}$. It can be shown that $\boldsymbol{g}$ is continuous, by $\inf _{a \in [\alpha _1,\alpha _2]} \det \{E(\boldsymbol{X} \boldsymbol{X}^T)\}(a) > 0$ and the fact that the sample paths of $X_j(a)$ is bounded by some constant τ. Therefore, by the continuous mapping theorem, $\boldsymbol{g}(\textrm{f}_{n}({\boldsymbol{E}}_n)) {\buildrel {d} \over \longrightarrow }\boldsymbol{g}(\boldsymbol{E})$ in $(\ell ^\infty ( [\alpha _1,\alpha _2]\setminus I_{\delta }))^{p}$ as $n \rightarrow \infty$. This and Eq. C14 imply

$$ \sqrt{n} f_n\left( \overline{\boldsymbol{Z}}\right) \left( a\right) {\buildrel {d} \over \longrightarrow }\boldsymbol{g}\left( \boldsymbol{E}\right) $$

in $(\ell ^\infty ( [\alpha _1,\alpha _2]\setminus I_{\delta }))^{p}$ as $n \rightarrow \infty$. This, Eqs. C13, and C8 then imply

$$ -2\log f_n\left( \mathcal {R}(\beta _j)\right) \left( a\right) {\buildrel {d} \over \longrightarrow }\boldsymbol{g}\left( \boldsymbol{E}\right) ^T \left( a\right) \boldsymbol{\sigma }^{-2}\left( a\right) \boldsymbol{g}\left( \boldsymbol{E}\right) \left( a\right) $$

in $$\ell ^\infty ( [\alpha _1,\alpha _2]\setminus I_{\delta })$$ as $$n \rightarrow \infty $$.

## Appendix D Proof of Corollary 2

Since $\hat{\boldsymbol{g}}\left( {\boldsymbol{E}}_n^*\right) \left( a\right)$ can be rewritten as

$$ \left\{ \sum _{i = 1}^n \left( \boldsymbol{X}_i \boldsymbol{X}_{i}^T\right) / n\right\} \left( a\right) {\boldsymbol{U}}_n^*\left( a\right) - \left\{ \sum _{i = 1}^n \left( \boldsymbol{X}_i \boldsymbol{X}_{i, (-j)}^T\right) / n\right\} \left( a\right) {\boldsymbol{U}}_{n, (-j)}^*\left( a\right) , $$

most of the components in $\hat{\Uppsi }^{*}(a) = \hat{\boldsymbol{g}}(\boldsymbol{E}_n^*)^T \hat{\boldsymbol{\sigma }}^{-2}(a) \hat{\boldsymbol{g}}(\boldsymbol{E}_n^*)$ have been studied in the previous Appendix sections. Thus, to study bootstrap consistency of $\textrm{f}_n(\hat{\Uppsi }^{*})(a)$, it suffices to show that $\textrm{f}_{n}(\text{ vec }(\hat{\boldsymbol{\sigma }}^{2} - {\boldsymbol{\sigma }}^{2})) = o_p(1)$. This, the uniform convergence of $\textrm{f}_{n}({\boldsymbol{\sigma }}^2)(a)$ to ${\boldsymbol{\sigma }}^2(a)$ in the second paragraph of Appendix C, Eq. A5, the bootstrap consistency of $\textrm{f}_n({\boldsymbol{U}}_n^*)(a)$ in Appendix B, and a routine extension of the proof for the conditional Slutsky’s lemma in Cheng (2015) to the case of random elements of a metric space, imply that $(\textrm{f}_n({\boldsymbol{U}}_n^*), \textrm{f}_{n}(\text{ vec }(\hat{\boldsymbol{\sigma }}^{2})), \textrm{f}_{n}\left( \sum _{i = 1}^n \text{ vec }\left( \boldsymbol{X}_{i} \boldsymbol{X}_{i}^T\right) / n\right) )^T$ is bootstrap consistent for $({\boldsymbol{U}}, \mathrm{ vec }({\boldsymbol{\sigma }}^2), \text {vec}\left\{ E\left( \boldsymbol{X} \boldsymbol{X}^T \right) \right\} )^T$ in $\{\ell ^\infty ([\alpha _1,\alpha _2]\setminus I_\delta )\}^{p(1+2p)}$. The desired result follows by the continuous mapping theorem for the bootstrap (see, e.g., Kosorok 2008b, Theorem 10.8).

The desired $\textrm{f}_{n}(\text{ vec }(\hat{\boldsymbol{\sigma }}^{2} - {\boldsymbol{\sigma }}^{2})) = o_p(1)$ is true due to the weak consistency of $\hat{\boldsymbol{\sigma }}^{2}$ for ${\boldsymbol{\sigma }}^{2}$ in $\ell ^\infty ([\alpha _1,\alpha _2])$ and the fact that $\sup _{a \in [\alpha _1, \alpha _2]} \left\| \textrm{f}_{n}(\text{ vec }(\hat{\boldsymbol{\sigma }}^{2} - {\boldsymbol{\sigma }}^{2}))(a)\right\| \le \sup _{a \in [\alpha _1, \alpha _2]} \left\| \text{ vec }(\hat{\boldsymbol{\sigma }}^{2} - {\boldsymbol{\sigma }}^{2})\right.$ $\left. (a)\right\|$. The consistency of $\hat{\boldsymbol{\sigma }}^{2}$ for ${\boldsymbol{\sigma }}^{2}$ can be obtained as follows. We can decompose $\hat{\boldsymbol{\sigma }}^{2}(a)$ as

D15 $$\begin{aligned} \frac{1}{n}\sum _{i=1}^n \left\{ \left( \boldsymbol{X}_i\varepsilon _i\right) \left( \boldsymbol{X}_i\varepsilon _i\right) ^T\right\} \left( a\right) + \frac{1}{n}\sum _{i=1}^n \left\{ \left( \boldsymbol{X}_i\boldsymbol{X}_i^T\right) \left( a\right) \left( \hat{\varepsilon }_i^2 - \varepsilon _i^2\right) \right\} . \end{aligned}$$

The first term can be shown to be consistent for ${\boldsymbol{\sigma }}^{2}(a)$ in $\ell ^\infty ([\alpha _1,\alpha _2])$ by Theorem S.1 of Chang and McKeague (2022b) and Lemma 9.28 of Kosorok (2008b). Each element of the second term of Eq. D15 can be shown to be $o_p(1)$ as follows, so that the desired consistency of $\hat{\boldsymbol{\sigma }}^{2}$ for ${\boldsymbol{\sigma }}^{2}$ can be obtained by the Slutsky’s lemma. We begin by noting that

D16 $$\begin{aligned} \hat{\varepsilon }_i^2 - \varepsilon _i^2 = 2Y_i\boldsymbol{X}_i^T({\boldsymbol{\beta }} - \hat{\boldsymbol{\beta }}) + \hat{\boldsymbol{\beta }}\boldsymbol{X}_i\boldsymbol{X}_i^T(\hat{\boldsymbol{\beta }}-{\boldsymbol{\beta }})+(\hat{\boldsymbol{\beta }}-{\boldsymbol{\beta }})\boldsymbol{X}_i\boldsymbol{X}_i^T{\boldsymbol{\beta }}. \end{aligned}$$

We can show $\beta _j(a)$ is bounded by some finite constant because it has bounded variation over $[\alpha _1, \alpha _2]$, which, together with the uniform boundedness of $X_j(a)$ and ε(a), imply the uniform boundedness of *Y*. This and the definition of $\hat{\boldsymbol{\beta }}$ in turn imply the uniform boundedness of $\hat{\boldsymbol{\beta }}$. These boundedness results, Eq. D16, and $\left| \hat{\boldsymbol{\beta }}-{\boldsymbol{\beta }}\right| = o_p(1)$ (by a similar reasoning as in the third paragraph of Appendix B) imply $\max _{i = 1, \ldots , n} \left| \hat{\varepsilon }_i^2 - \varepsilon _i^2\right| = o_p(1)$. Therefore, each element of the second term of Eq. D15 can be bounded by $\tau ^2 \max _{i = 1, \ldots , n} \left| \hat{\varepsilon }_i^2 - \varepsilon _i^2\right| = o_p(1)$.

## Code Availability

Upon request.
